# Supplementary material for: Optimized ChIP-seq method facilitates transcription factor profiling in human tumors
Source: Life Sci Alliance. 2018 Dec 28;2(1):e201800115. doi: 10.26508/lsa.201800115 (PMC6311467; doi:10.26508/lsa.201800115)
Supplement: Supplementary file 1 [file LSA-2018-00115_TableS1.docx]

|  | **p#** | **tumor** | **grade/gleason** | **HR status** | **tumor cell%** | **tissue block dimensions** | **cryosections per IP** |
| --- | --- | --- | --- | --- | --- | --- | --- |
| **breast** | M1 | invasive ductal carcinoma | grade 3 | ER+ PR+ HER2+ | 65% | 7 mm x 2 mm | 17 x 30µ |
|  | M2 | infiltrating ductal carcinoma | grade 2 | ER+ PR+ HER2- | 75% | 4 mm diameter | 16 x 30µ |
|  | M3 | ductal carcinoma in-situ (DCIS) | - | ER+ PR+ HER2- | 80% | 5 mm diameter | 15 x 30µ |
|  | M4 | infiltrating ductal carcinoma | grade 4 | ER+ PR+ HER2- | 80% | 4,5 mm x 5,5 mm | 10 x 30µ |
| **prostate** | P1 | prostate adenocarcinoma | pT3aN0Mx / 9 | AR+ | 65% | 5 mm x 4 mm | 20 x 30µ |
|  | P2 | prostate adenocarcinoma | pT2cN0Mx / 7 | AR+ | 70% | 6 mm x 3 mm | 20 x 30µ |
|  | P3 | prostate adenocarcinoma | cT2bN0Mx / 10 | AR+ | 65% | 3,5 mm x 4 mm | 18 x 30µ |
|  | P4 | prostate adenocarcinoma | pT3aN0Mx / 9 | AR+ | 70% | 3,5 mm x 6 mm | 12 x 30µ |
|  | biopsy 1 | prostate adenocarcinoma | pT3aN0Mx / 7 | AR+ | 70% | 18G biopsy, 3 mm long | NA |
|  | biopsy 2 | prostate adenocarcinoma | pT3aN0Mx / 7 | AR+ | 60% | 18G biopsy, 5 mm long | NA |
| **endometrium** | E1 | endometroid adenocarcinoma | I (Figo) | ER+ | 70% | 7 mm x 5,5 mm | 15 x 30µ |
|  | E2 | endometroid adenocarcinoma | I (Figo) | ER+ | 70% | 10 mm x 7,5 mm | 14 x 30µ |
|  | E3 | endometroid adenocarcinoma | I (Figo) | ER+ | 90% | 9 mm x 6 mm | 8 x 30µ |

**Supplementary Table S1:** details in clinical samples and amount of tissue used for ChIP-seq
