# Supplementary material for: Optimized ChIP-seq method facilitates transcription factor profiling in human tumors
Source: Life Sci Alliance. 2018 Dec 28;2(1):e201800115. doi: 10.26508/lsa.201800115 (PMC6311467; doi:10.26508/lsa.201800115)
Supplement: Supplementary file 2 [file LSA-2018-00115_TableS2.docx]

**Supplementary Table S2**: primer sequences used in this study

| **Primers ChIP qPCRs** |  |  |
| --- | --- | --- |
|  | Forward primer | Reverse primer |
| negcon_1 | TGCCACACACCAGTGACTTT | ACAGCCAGAAGCTCCAAAAA |
| negcon_2 | CTAGGAGGGTGGAGGTAGGG | GCCCCAAACAGGAGTAATGA |
| negcon_3 | TGGCCCTTGATACTGGAGTC | GACATCCAAGGCAAGATGGT |
| negcon_4 | GCTGCTTTGGGAAATGGTTA | TGGTCGCAGGTTTCAACATA |
| RARA_1 | CGCTGGGAACTTCTGTTCTT | AGGGCAGCTCAGATAACAGG |
| RARA_2 | GCTGGGTCCTCTGGCTGTTC | CCGGGATAAAGCCACTCCAA |
| XBP1_1 | ATACTTGGCAGCCTGTGACC | GGTCCACAAAGCAGGAAAAA |
| XBP1_2 | TCTCTGGGCTGGCACCAT | GCGGTGCGTAGTCTGGAG |
| XBP1_3 | CGGAGGACTAAGTGCTAGGC | GCTGAAATAACAGAGCTGTTGAAA |
| GREB1 | CAGCTGACTGTCTTCCACCA | AGCCCTGAAGTGTTTTGCTG |
| KLK3_enh | GCCTGGATCTGAGAGAGATATCATC | ACACCTTTTTTTTTCTGGATTGTTG |
| KLK2 | AGCATCTAGGTGCCAACAGG | GACAAGGCGATGGAGAGAAC |
| DDIT4 | CTAGAGCTCGCGGTCTGGTCTGGTCT | GGCGAAGAGGAGGTGGACGACGACGAGAAG |
| ARpeak_chr12 | TGTGTCCCTGTTCTCAAGGG | CCCACTTTTGTTTGCCGAGG |
| CUX2 | GTTGATAGGCGGGTCTGGTG | CCCTCTGTCCCCTTGTTCTC |
| EEF2K | GCCCTTGCATGATGACAGTTT | TGCAGAAGCCAGAGATACGTC |
| ZBTB16 | CAGTGTGCTGTTCTCCGTCT | CAGACGCAGGGCATTTTACA |
| UBC | GTAATGACGGGGCTTCCTTT | TGAGATCTGCCGAGTCATTG |
|  |  |  |
| **Primers differential sites FA vs DSG/FA H3K27ac ChIP qPCR** | |  |
|  | Forward primer | Reverse primer |
| **MCF7/mamma tissue** |  |  |
| shared_1 (=RARA_1) | CGCTGGGAACTTCTGTTCTT | AGGGCAGCTCAGATAACAGG |
| shared_2 | CCAGAGAGTCGTGGCGTTTC | TCTGAGCGCAGCCTGTCTTA |
| DSG only | AGGGTTCAACTCAAGGGAATCAC | TGCACAATACCATCTCTCACAGT |
| DSG only_2 | CACCCACCTCGCAGAATTGC | CTGGAGCCAGGATGTGAGCA |
| FA only | CCCACTCACTTGCTGTGTGA | TGGTGGACAACCTGGTTTGG |
| negcon_3 | TGGCCCTTGATACTGGAGTC | GACATCCAAGGCAAGATGGT |
| **LNCaP/prostate tissue** |  |  |
| shared_1 (=DDIT4) | CTAGAGCTCGCGGTCTGGTCTGGTCT | GGCGAAGAGGAGGTGGACGACGACGAGAAG |
| shared_2 | AGCACAGGTAGGTTCCGCTC | GAGTGGGCTCGGTTCGAGAT |
| DSG only | GTTCCTGTCTGGCTCAAGGTTA | GTGCCTGTAGAGCCGAGTC |
| DSG only_2 | CTCCATCAAATTACTCTGTCCAGC | CAAGGCTTGGGAAAGAGAGAATAAA |
| FA only | TGGGCTTTACTGAGGGGTGATT | GGATGTCTGACCAGGCTATGAG |
| negcon_4 | GCTGCTTTGGGAAATGGTTA | TGGTCGCAGGTTTCAACATA |
| **endometrial tissue** |  |  |
| shared_1 | AGTCAGACACTCTTGCGGCT | CTGGACCTCGCTGATTCGTG |
| shared_2 | AGGGGAGACCTTTGGCCTAC | CTCTGACGCTTATCGACGCC |
| DSG only | GACCACTCTGCGGTCTACCC | CAAGGATAAGGCGCGAGGC |
| FA only | TCAGATAGCCCCACCGAAGG | GTGTTGGCCCCTCTATGAGC |
| negcon_2 | CTAGGAGGGTGGAGGTAGGG | GCCCCAAACAGGAGTAATGA |
